# Impact of the FTO Gene Variation on Appetite and Fat Oxidation in Young Adults

**DOI:** 10.3390/nu15092037

**Published:** 2023-04-23

**Authors:** Jesús G. Ponce-Gonzalez, Ángel Martínez-Ávila, Daniel Velázquez-Díaz, Alejandro Perez-Bey, Félix Gómez-Gallego, Alberto Marín-Galindo, Juan Corral-Pérez, Cristina Casals

**Affiliations:** 1 ExPhy Research Group, Department of Physical Education, Instituto de Investigación e Innovación Biomédica de Cádiz (INiBICA), Universidad de Cádiz, 11519 Cádiz, Spain; jesusgustavo.ponce@uca.es (J.G.P.-G.);; 2 Faculty of Nursing Salus Infirmorum, University of Cádiz, 11001 Cádiz, Spain; 3 AdventHealth Research Institute, Neuroscience Institute, Orlando, FL 32804, USA; 4 GALENO Research Group, Department of Physical Education, Faculty of Education Sciences, University of Cádiz, Puerto Real, 11519 Cádiz, Spain; alejandro.perezperez@uca.es; 5 Faculty of Health Sciences, University Rey Juan Carlos, 28922 Alcorcón, Spain

**Keywords:** metabolism, physical exercise, genetic association studies, lipid utilization

## Abstract

The FTO rs9939609 gene, which presents three polymorphisms (AA, AT, and TT), has been associated with the development of obesity through an increased fat accumulation; however, the associations of the gene with other physiological mechanisms, such as appetite or fat oxidation, are still unclear. Therefore, this study aims to evaluate the influence of the FTO rs9939609 gene on different obesity-related factors in young adults. The FTO rs9939609 polymorphism was genotyped in 73 participants (28 women, 22.27 ± 3.70 years). Obesity-related factors included dietary assessment, physical activity expenditure, body composition, appetite sensation, resting metabolic rate, maximal fat oxidation during exercise (MFO), and cardiorespiratory fitness. Our results showed that TT allele participants expressed higher values of hunger (*p* = 0.049) and appetite (*p* = 0.043) after exercising compared to the AT allele group. Moreover, the TT allele group showed significantly higher values of MFO (*p* = 0.031) compared to the AT group, regardless of sex and body mass index. Thus, our results suggest that the FTO rs9939609 gene has an influence on appetite, hunger, and fat oxidation during exercise, with TT allele participants showing significantly higher values compared to the AT allele group. These findings may have practical applications for weight loss and exercise programs.

## 1. Introduction

Obesity can be defined as an excessive accumulation of fat that may generate deleterious effects on health, increasing the risk of suffering from metabolic or cardiovascular diseases [1]. Over the past 50 years, the prevalence of overweight and obesity has risen to the point where it is considered a global pandemic [2]. For this reason, in recent years the literature has tried to analyze the relevant factors associated with the condition to search for preventive strategies. Obesity is a condition affected by numerous factors, from psychological problems to an unhealthy lifestyle, which can include incorrect dietary habits or a lack of physical activity. In addition to this, genetics also plays a role in the development of overweight and obesity [3].

Among all the human genomes, several genes have been associated with overweight and obesity, and the fat mass and obesity-associated gene (FTO), which is encoded on chromosome 16, has been one of the most investigated in the prevention of obesity [4]. Several single-nucleotide polymorphisms of the FTO have been associated with increased levels of body mass index (BMI), hip circumference, total body mass, and fat mass [4,5]. Specifically, the FTO rs9939609 gene, which presents three polymorphisms (AA, AT, and TT), has been closely associated with the development of obesity [6,7]. Particularly, the A allele has been known as the risk allele with homozygous carriers of the A allele having a higher weight than non-carriers in middle age adults [8] and higher values of body mass index and fat mass in premenopausal women [9] compared to T allele participants. However, the underlying mechanism of why this allele may promote obesity is still unclear. It has been proposed that the AA allele might alter the suppression of postprandial appetite, increasing the preference for high-calorie dense foods, stimulating hyperphagia and modulating satiety in the hypothalamus [6,10]. Nevertheless, there is still controversy to this proposal because other authors have not found any significant differences in the regulation of appetite of the FTO rs9939609 gene [11]; therefore, other physiological explanations should be searched for. One physiological mechanism in which the FTO rs9939609 gene could modulate fat mass is through the alteration of energetic metabolism and fat oxidation [12,13].

The ability to oxidize fat during exercise, also known as maximal fat oxidation (MFO), has been proposed as a preventive factor against several cardiometabolic factors, such as insulin resistance, body mass, plasma triglycerides, and obesity [14,15]. For this reason, physical exercise training focused on improving MFO could provide a safe and practical method in the treatment and prevention of obesity. Nonetheless, the influence of the FTO gene on resting fat oxidation has shown contradictory results [12,13] and, to our knowledge, the association between the FTO rs9939609 gene and MFO has not been studied yet.

Thus, this study aimed to investigate the influence of the FTO rs9939609 gene on different obesity-related factors in young adults, such as dietary habits, physical activity, body composition, appetite sensation, resting metabolic rate, MFO, and cardiorespiratory fitness.

## 2. Materials and Methods

### 2.1. Design

This study, with a cross-sectional design, is part of the NutAF research project [15,16,17,18,19]. All participants gave their written informed consent to participate after being fully informed of the objectives of the study and its possible side effects. The study was approved by the Hospital Puerta del Mar Ethical Committee (Cádiz, Spain) and followed the Helsinki declaration.

After participants gave their informed written consent, they were provided with a 5-day dietary record and an accelerometer to register both diet and physical activity and were also told to maintain their usual lifestyle. After 7 days of fasting, blood samples were obtained to assess the FTO gene rs9939609 polymorphism and participants returned their dietary records and accelerometers. They performed anthropometric and body composition assessments (electrical bioimpedance), appetite scales, basal metabolism tests, and physical exercise tests to determine MFO and maximal oxygen consumption (VO_2max_). All evaluations were performed in the morning (between 8–10 h AM) and in a fasted state. Participants were also told to avoid vigorous physical activity and prevent alcohol and caffeine intake the day before the evaluations. 

### 2.2. Participants

In the present study, a total of 73 young adults (28 women, 22.27 ± 3.70 years old) were recruited. Inclusion criteria included the following: (i) aged between 18 and 35 years and (ii) maintaining a stable body mass in the last 6 months. Exclusion criteria were as follows: (i) being an active smoker, (ii) having any cardiovascular disease, injury, or any other condition that may prevent them from performing physical activity, and (iii) having a vitamin supplementation in the 6 preceding months. 

### 2.3. Dietary Assessment

The dietary assessment was evaluated using a 5-consecutive-day dietary record (including 2 weekend days) by weighting. Participants were told to maintain their usual intakes and were provided with a digital scale if they did not have one. Energy intakes (kcal) and macro- and micronutrients of the dietary records were analyzed using the DIAL software (version 1.19). 

### 2.4. Physical Activity

To estimate physical activity, we utilized a triaxial ActiGraph GT3X+ accelerometer (ActiGraphInc, Pensacola, FL, USA) that was securely attached to the back of the hip [20] for seven consecutive days, except for water-based activities and sleep. The placement of the accelerometer at the hip accurately permitted the assessment of sedentary behaviour [21]. 

Raw data from the accelerometer were collected at a measurement rate of 100 Hz and downloaded in 60 s epochs [22]. Data were considered valid if recorded for ≥10 h per day on at least three weekdays and one weekend day [22]. Wear-time validation was performed using Choi’s algorithm with default settings [23]. 

To estimate the daily energy expenditure of all participants, their physical activity was categorized into three intensities: light physical activity (LPA, 150–2690 counts/min), moderate physical activity (MPA, 2691–6166 counts/minute), and vigorous physical activity (VPA, 6167–9642 counts/minute) [24] 

### 2.5. Blood Samples

As part of the study protocol, fasting blood samples were collected from the participants’ antecubital vein using EDTA tubes. After collection, the blood samples were stored at −80 °C until they could be further analyzed. 

Triglyceride and glucose and plasma levels were determined using Spinreact commercial kits (TAG, ref. 10013110; glucose-HK, ref. 1001200) according to the manufacturer’s instructions. The intra-assay coefficients of variation were <0.4% and <1%, while the inter-assay coefficients of variation were <3.6% and <1.5% for triglycerides and glucose, respectively. Absorbance readings were taken using a BIO-TEK PowerWaveTM 340 microplate reader and analyzed with the BIO-TEK KC JuniorTM program (Bio-Tek Instruments Inc., Winooski, VT, USA).

Additionally, we used a MILLIPLEX^®^ MAP Human Metabolic Hormone Magnetic Bead Panel (HMHEMAG-34K, Millipore Sigma, Burlington, MA, USA) and Luminex^®^ 200TM System (Luminex Corp., Austin, TX, USA) to measure plasma insulin, ghrelin, leptin, gastric inhibitory peptide (GIP), Glucagon-like peptide-1 (GLP-1), and Peptide YY (PYY) levels, following the manufacturer’s instructions. To convert insulin data from pg/mL to mUI/L, we followed the method previously described [25]. The intra-assay coefficients of variation for insulin and leptin were less than 10%, and the inter-assay coefficients of variation were less than 15%.

To determine the polymorphism of the FTO rs9939609 gene, DNA from the blood samples was obtained using the High Pure PCR Template Preparation commercial kit (Roche Applied Science, Mannheim, Germany). The FTO rs9939609 gene was genotyped in the entire sample using Custom TaqMan^®^ SNP (single nucleotide polymorphism) Genotyping Assays (C_30090620_10, Applied Biosystems, Foster City, CA, USA) consisting of preoptimized Polymerase chain reaction (PCR) primer pairs and two probes for allelic discrimination. 

The amplification of PCR was performed using a StepOne™ Real-Time PCR (Life Technologies, Foster City, CA, United States) consisting of a first 10 min phase of denaturation at 95 °C, 50 cycles of denaturation at 92 °C during 15 s, annealing/extension at 60 °C during 1 min, and a final extension phase at 60 °C during 30 s. 

### 2.6. Body Composition

Height was registered in a standing position (SECA 225, range 60–200 cm; precision of 1 mm). Body mass (kg), body fat (kg), and lean body mass (kg) were estimated through a multifrequency 8-point electrode bioelectrical bioimpedance (TANITA-MC780MA, Barcelona, Spain), following the manufacturer’s instructions. Body mass index was calculated by dividing body mass in kilograms by squared height in meters. 

### 2.7. Appetite Sensation

Before and immediately after the MFO and VO_2max_ tests, participants completed a subjective appetite rating measured by a 10 cm visual analogue scale. This scale included ratings of hunger, satiety, fullness, and prospective food consumption [26]. Depending on the participant’s answers, general appetite and a general fullness ratio were calculated. 

### 2.8. Resting Metabolic Rate

The resting metabolic rate (kcal) was estimated through indirect calorimetry using a Jaeger MasterScreen CPX^®^ (CareFusion, San Diego, JA, USA) in a conditioned room (21 ± 1 °C, 50 ± 2% relative humidity) for 30 min. Then, a stable period of 5 min with a coefficient of variation for oxygen uptake and carbon dioxide production lower than 15% was used to calculate the resting metabolic rate using Frayn’s equation [27]. 

Then, the daily energy balance was calculated as the dietary energy intake minus total energy expenditure (physical activity plus resting metabolic rate). 

### 2.9. Maximal Fat Oxidation and Cardiorespiratory Fitness

The MFO test (an incremental test with 3 min steps at a constant pedaling of 60–80 rpm) was performed on a cycle ergometer (Lode Excalibur, Groningen, Netherlands) with 15 W increments in overweight/obese participants and 30 W in normal-weight participants until the respiratory exchange ratio hit 1 [28]. After a 5 min recovery period, the VO_2max_ test was performed [28], beginning at the load at which the MFO test ended. The VO_2max_ test consisted of a 1 min incremental test until exhaustion, at the same load rate and cadence as described for the MFO test. 

Fat oxidation was estimated using VO_2_ and CO_2_ averages of the last 60 s of each step using the aforementioned Frayn’s equation [27]. The percentage of VO_2max_ in each step was similarly obtained, with the VO2 averages of the last 60 s. Then, using these values, a polynomial curve that best fits the present analysis was drawn. Then, MFO was expressed as absolute values (g/min) and also relativized to cardiorespiratory fitness (MFO/VO_2max_, mg/mL/min) and to fat mass (MFO/lean mass, mg/kg/min).

### 2.10. Statistical Analysis

Unless otherwise specified, data are presented as mean ± standard deviation (SD). The normality of distribution, homogeneity of variance, and sphericity were assessed using the Kolmogorov–Smirnov, Levene, and Mauchly tests, respectively.

An independent samples t-test was utilized to evaluate the differences between males and females. A chi-square test and a one-way analysis of variance (ANOVA) followed by Bonferroni post hoc comparisons, were performed to study differences between the FTO rs9939609 polymorphisms (AA, AT, and TT). In addition to this, to study the changes in appetite and hunger sensations after the MFO and VO_2max_ tests, a mixed factorial ANOVA was performed to detect significant differences. Finally, to study the possible influence of sex and BMI on MFO, a factorial ANOVA was applied when the analysis included more than one independent variable (FTO, sex, BMI). 

All analyses were carried out using IBM SPSS Statistics version 26 software (SPSS Inc., Chicago, IL, USA) with a significance level of *p* < 0.05.

## 3. Results

Table 1 displays sexual differences. Men had higher values than women in energy intake, plasma glucose, height, lean mass, resting metabolic rate, energy expenditure, and maximal oxygen consumption. In contrast, women had higher values than men in ghrelin, leptin, and absolute and relative fat mass (*p* < 0.05).

The general characteristics of the FTO gene polymorphisms are shown in Table 2. There were significant differences in vigorous physical activity between the AA and TT groups, with the homozygotes spending less time in this physical activity behaviour. For the rest of the variables, there were no significant differences.

**Figure 1:**
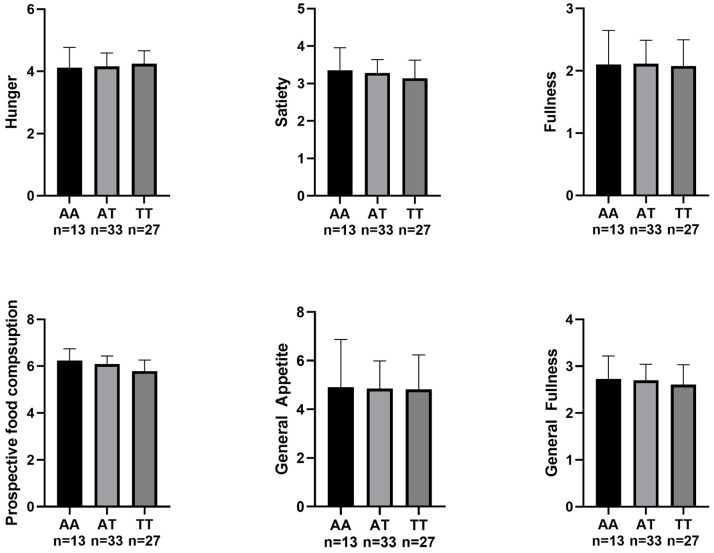
Participants’ appetite sensation before the MFO and VO_2max_ tests.

**Figure 2:**
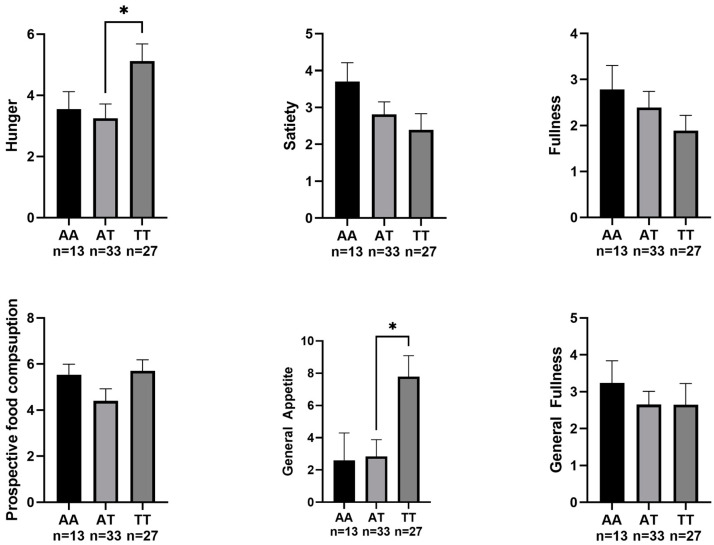
Participants’ appetite sensation after the MFO and VO_2max_ tests. * *p* < 0.05.

Regarding appetite sensation, there were no significant differences in pre-exercise appetite sensation in any of the FTO polymorphisms (Figure 1). After performing the exercise tests, significant differences were only found in appetite sensation between the different FTO polymorphisms, specifically in hunger (F (2,71) = 3.109, *p* = 0.049, η^2^ = 0.081) and general appetite (F (2,71) = 3.279, *p* = 0.043, η^2^ = 0.085). Bonferroni post hoc comparisons are shown in Figure 2.

A significant principal effect was found between FTO polymorphism and absolute MFO (F (2,61) = 3.69, *p* = 0.031, ηp^2^ = 0.108), MFO/VO_2max_ (F (2,61) =6.04, *p* = 0.004, ηp^2^ = 0.165), and MFO/lean mass (F (2,61) = 4.87, *p* = 0.011, ηp^2^ =0.138). Bonferroni’s post hoc comparisons between FTO polymorphisms (AA, AT, and TT) are shown in Table 3. 

No significant interactions were found between the FTO polymorphism, sex, and BMI in any of the MFO values studied (*p* > 0.05). Similar to this, no significant interactions were found between FTO polymorphisms and sex or FTO polymorphisms and BMI in any of the MFO values (*p* > 0.05).

## 4. Discussion

The main findings of this cross-sectional study are that the FTO rs9939609 gene is associated with postexercise appetite sensation and fat oxidation in young adults, regardless of sex or BMI. In this study, the FTO rs9939609 TT genotype showed higher values of hunger and appetite after exercise. In addition to this, the AT carriers showed lower values of fat oxidation compared to TT genotypes, showing a decreased MFO compared to the TT polymorphism.

The FTO rs9939609 gene has been considered an obesity gene, with adults with the AA allele showing higher values in weight and BMI than adults who carried the T allele [29]. Nevertheless, no significant differences were found for any of the anthropometric or body composition variables in our sample. However, it is worth mentioning that we found significant differences between the genotypes, as participants with the AA allele had higher values of body mass, BMI, and fat mass than those who carried the T allele. Similar to our findings, a previous study did not find any significant differences between FTO polymorphisms in obese women, but they also observed clinically significant differences [9]. No significant differences were found for energy expenditure or cardiorespiratory fitness between the alleles. Nonetheless, there were a trend to signification in energy intake and daily energy balance, with the TT allele group showing a higher daily energetic intake and daily energy balance than the participants who carried the A allele. 

In addition to this, the present study suggests an association of the FTO rs9939609 polymorphism with postexercise appetite sensation. In our study, the TT allele of the FTO rs9939609 gene had significantly higher values of hunger and overall appetite than the AT allele. To our knowledge, only one article evaluated appetite sensation after an acute exercise bout of exercise, and no significant differences were found between AA and TT allele participants, similar to our data [30]. 

However, it is worth mentioning that our data showed a trend towards signification between these two groups (*p* = 0.070). In line with this, previous authors have shown that appetite sensation appeared to be elevated in the TT allele compared to the AT allele. Magno et al. [30] showed that appetite sensation appeared early in TT allele participants compared to AT allele participants after consuming an isocaloric menu, showing higher values at 30, 60, and 150 min after the intake in a sample of 70 women. Another study including both men and women published similar results, with participants with the AA or AT alleles showing lower values of postprandial hunger than the TT allele participants [31]. 

Previous literature has associated the FTO rs9939609 gene with an increased risk of adiposity and type 2 diabetes [29]. Type 2 diabetes and obesity have been related to low-fat oxidation rates [14]. A better ability to oxidize fat during exercise has been associated with lower metabolic disease risk in overweight sedentary men [32], better insulin sensitivity [33], and lower cardiometabolic risk in young adults [15]. Consistent with previous research, the data from this study suggest that participants with the at-risk allele had lower fat oxidation values during exercise, especially those with the AT genotype, regardless of sex, BMI, or cardiorespiratory capacity. To our knowledge, this is the first study that has found an association between the ability to oxidize fat during exercise and the FTO rs9939609 gene, and this association remains regardless of the sex and BMI. The presence of the TT allele in the FTO rs9939609 gene appears to provide better metabolic capacity and flexibility, potentially serving as a protective factor against obesity and cardiometabolic diseases. 

One possible explanation for the higher levels of hunger in the TT group may be explained by the results of this study. TT allele participants have shown a higher capacity to oxidize fat during exercise, both in absolute and relative values, which could generate a hormonal response to increase hunger and appetite and be able to restore the energetic reserves to their baseline values. This could also explain why the TT allele carriers showed a higher (with a trend towards signification) daily energy balance compared to the other two alleles, even though the three alleles showed a similar energy expenditure, suggesting that exercise may exert a hunger response in these participants. Nevertheless, this study has not included any postexercise hormonal markers, so this explanation is only a hypothesis. In addition to this, the influence of the FTO rs9939609 gene on appetite and hunger sensation is still not clear, with some authors showing that TT allele participants had lower values of postprandial hunger than carriers of the A allele [6]; therefore, more studies are needed to clarify the possible influence of the gene on hunger and appetite both at resting and in postexercise conditions. 

It should be noted that this study is not without limitations. Firstly, due to the cross-sectional design, we cannot establish cause–effect relationships between the outcomes. Moreover, the number of participants included limited the ability to generalize the outcomes since all of our subjects were young, apparently healthy (despite being overweight/obese), and living in the province of Cadiz (south of Spain). Notwithstanding, the post hoc power calculations for one of our main studied variables (MFO/lean mass) was 0.98. Nonetheless, a multicenter study including the different provinces of the region is encouraged to compare our results to those obtained from other populations. Furthermore, participants were excluded if they were smokers or had any cardiovascular disease, among others, potentially compromising the external validity of the results. Therefore, future studies should include a larger cohort with a longitudinal design, also prioritizing results obtained through genome-wide association studies. 

However, a strength of this study is the assessment of MFO and VO_2max_ using an exercise protocol test and adequate equipment under laboratory-controlled conditions. In addition to this, not only have we included the energy intakes of the diet but also accurate energy expenditure combining the data from the resting metabolic rate (measured through indirect calorimetry) and physical activity (measured through accelerometers), which allowed us to estimate a proper daily energetic balance that could be more interesting than analyzing only the energetic intakes or expenditures separately. Likewise, this study provides novel results, since to our knowledge no study to date has investigated the relationship between FTO rs9939609 polymorphism and fat oxidation during exercise and appetite in young adults.

## 5. Conclusions

The results of our study suggest that the FTO rs9939609 gene may have an impact on appetite and hunger after exercise, with the TT allele group expressing higher values compared to A allele carriers. Additionally, FTO rs9939609 polymorphism is associated with maximal fat oxidation (both in absolute and relative values) regardless of sex and BMI. Participants with the TT allele also demonstrated higher values of fat oxidation during exercise compared to those who were A allele carriers. These findings suggest that individuals with TT allele homozygotes may have better protection against cardiometabolic diseases due to their higher metabolic capacity and flexibility.

These factors may be relevant for designing exercise programs focused on weight loss in individuals with the homozygous T alleles, and interventions can be tailored accordingly to achieve optimal results.

## Figures and Tables

**Table 1:** Participants’ characteristics and differences between sexes.

	Men (*n* = 46)	Women (*n* = 27)	*p*
Men, No. (%)			
Age (years)	22.24 ± 3.67	23.18 ± 5.06	0.359
Energy intake (kcal/day)	2848.00 ± 1070.62	2233.32 ± 604.43	**0.007**
Light physical activity (min/day)	1911.48 ± 377.10	2104.48 ± 519.16	0.084
Moderate physical activity (min/day)	301.71 ± 96.70	332.72 ± 143.48	0.295
Vigorous physical activity (min/day)	38.33 ± 37.29	30.08 ± 27.01	0.407
Physical activity expenditure (kcal/day)	327.72 ± 132.67	289.56 ± 117.37	0.239
Plasma triglycerides (mg/dL)	71.72 ± 24.92	66.83 ± 24.58	0.414
Plasma glucose (mg/dL)	103.44 ± 9.64	97.03 ± 9.93	**0.008**
Plasma insulin (mUI/L)	23.56 ± 22.76	22.64 ± 19.37	0.860
Plasma ghrelin (pg/mL)	15.66 ± 9.31	25.27 ± 20.94	**0.009**
Plasma leptin (ng/mL)	1.99 ± 2.86	7.36 ± 4.93	**<0.001**
Plasma GIP (pg/mL)	65.98 ± 36.88	66.58 ± 29.38	0.942
Plasma GLP-1 (pg/mL)	8.45 ± 5.75	11.49 ± 9.16	0.085
Plasma PYY (pg/mL)	88.92 ± 45.48	94.44 ± 45.36	0.663
Height (cm)	176.74 ± 6.13	164.62 ± 6.81	**<0.001**
Body mass (kg)	78.75 ± 72.27	72.2 ± 16.98	0.089
BMI (kg/m^2^)	25.16 ± 4.17	26.86 ± 7.39	0.210
Fat mass (kg)	15.84 ± 9.26	23.37 ± 12.89	**0.005**
Fat mass (%)	18.95 ± 7.36	30.28 ± 9.54	**<0.001**
Lean mass (kg)	59.46 ± 6.49	46.04 ± 4.86	**<0.001**
Lean mass (%)	76.56 ± 6.55	65.54 ± 8.49	**<0.001**
Resting metabolic rate (kcal/day)	2013.29 ± 276.76	1580.07 ± 235.81	**<0.001**
Energy expenditure (kcal/day)	2321.49 ± 333.76	1838.60 ± 308.04	**<0.001**
Energy balance (kcal/day)	513.39 ± 1173.05	394.72 ± 703.76	0.630
VO_2max_ (mL/min)	3483.73 ± 530.92	2287.89 ± 419.47	**<0.001**
Vo_2max_ (mL/kg/min)	45.51 ± 10.55	33.72 ± 10.36	**<0.001**

Unless otherwise noted, values are expressed as mean ± standard deviation; Significant differences appear in bold; BMI, body mass index; VO_2max_, maximal oxygen consumption; GIP, gastric inhibitory peptide; GLP-1, Glucagon-like peptide-1; PYY, Peptide YY.

**Table 2:** Participants’ characteristics and differences between FTO gene polymorphisms.

	AA (*n* = 13)	AT (*n* = 33)	TT (*n* = 27)	*p*
Men, No. (%)	9 (69.2)	22 (64.7)	15 (55.6)	0.647
Age (years)	22.54 ± 4.48	22.15 ± 3.42	23.19 ± 5.07	0.642
Energy intake (kcal/day)	2474.00 ± 589.60	2412.00 ± 646.30	2941.00 ± 1323.00	0.086
Light physical activity (min/day)	1946.00 ± 444.60	1971.00 ± 415.00	1977.00 ± 470.7	0.978
Moderate physical activity (min/day)	328.30 ± 84.46	315.40 ± 104.10	305.40 ± 144.90	0.856
Vigorous physical activity (min/day)	9.22 ± 7.71 *	28.07 ± 22.07	33.38 ± 30.88	0.055
Physical activity expenditure (kcal/day)	365.30 ± 140.10	297.60 ± 121.80	307.60 ± 127.20	0.292
Plasma triglycerides (mg/dL)	76.39 ± 33.81	69.44 ± 24.37	67.26 ± 20.21	0.551
Plasma glucose (mg/dL)	99.54 ± 7.77	103.44 ± 10.49	98.66 ± 10.42	0.161
Plasma insulin (mUI/L)	16.66 ± 11.56	23.39 ± 21.48	26.14 ± 24.67	0.428
Plasma ghrelin (ng/mL)	21.88 ± 17.59	21.15 ± 18.22	15.92 ± 9.61	0.352
Plasma leptin (ng/mL)	5.31 ± 5.22	3.23 ± 3.10	4.12 ± 4.02	0.392
Plasma GIP (pg/mL)	74.36 ± 34.09	66.16 ± 6.91	62.35 ± 25.96	0.584
Plasma GLP-1 (pg/mL)	10.93 ± 7.15	9.39 ± 8.33	9.31 ± 6.20	0.798
Plasma PYY (pg/mL)	75.75 ± 33.17	96.16 ± 45.10	90.98 ± 50.58	0.478
Height (cm)	175.00 ± 9.34	3.23 ± 3.92	4.12 ± 4.23	0.177
Body mass (kg)	81.66 ± 15.66	75.15 ± 16.42	75.16 ± 15.34	0.412
BMI (kg/m^2^)	26.77 ± 5.40	25.00 ± 4.29	25.36 ± 4.95	0.518
Fat mass (kg)	19.86 ± 10.82	17.16 ± 10.31	18.53 ± 10.28	0.330
Fat mass (%)	23.25 ± 9.90	21.70 ± 9.05	22.73 ± 8.87	0.842
Lean mass (kg)	58.40 ± 9.04	54.65 ± 9.36	52.12 ± 7.46	0.104
Lean mass (%)	72.45 ± 9.06	73.68 ± 8.13	71.67 ± 9.09	0.454
Resting metabolic rate (kcal/day)	1978.00 ± 352.30	1801.00 ± 357.80	1840.00 ± 292.90	0.275
Energy expenditure (kcal/day)	2346.00 ± 467.90	2063.00 ± 370.30	2125.00 ± 378.30	0.102
Energy balance (kcal/day)	127.80 ± 782.40	317.00 ± 708.70	816.00 ± 253.80	0.066
VO_2max_ (mL/min)	3189.00 ± 680.00	2969.00 ± 674.60	3015.00 ± 904.40	0.680
Vo_2max_ (mL/kg/min)	39.92 ± 8.97	40.59 ± 10.64	42.00 ± 14.63	0.849

Unless otherwise noted, values are expressed as mean ± standard deviation; Significant differences appear in bold; BMI, body mass index; VO_2max_, maximal oxygen consumption, GIP, gastric inhibitory peptide; GLP-1, Glucagon-like peptide-1; PYY Peptide YY. * Differences with the TT group.

**Table 3:** Differences in MFO between FTO gene polymorphisms.

	AA (*n* = 13)	AT (*n* = 33)	TT (*n* = 27)	*p*
MFO (g/min)	0.38 ± 0.15	0.33 ± 0.15 *	0.42 ± 0.15	**0.031**
MFO/VO_2max_ (mg/mL/min)	0.12 ± 0.04	0.11 ± 0.04 **	0.14 ± 0.04	**0.004**
MFO/lean mass (mg/kg/min)	6.57 ± 2.37	6.25 ± 2.86 *	8.21 ± 2.76	**0.011**
Fatmax (%)	40.98 ± 8.81	40.25 ± 7.31	42.07 ± 6.88	0.642

Values are expressed as mean ± standard deviation; Significant differences appear in bold; MFO, maximal fat oxidation; VO_2max_, maximal oxygen consumption; * *p* < 0.05, ** *p* < 0.02, compared to the TT allele.

## Data Availability

The data presented in this study are available on reasonable request from the corresponding authors.
